# Y-Linked Copy Number Polymorphism of Target of Rapamycin Is Associated with Sexual Size Dimorphism in Seed Beetles

**DOI:** 10.1093/molbev/msad167

**Published:** 2023-07-22

**Authors:** Philipp Kaufmann, R Axel W Wiberg, Konstantinos Papachristos, Douglas G Scofield, Christian Tellgren-Roth, Elina Immonen

**Affiliations:** Department of Ecology and Genetics (Evolutionary Biology program), Uppsala University, Uppsala, Sweden; Department of Ecology and Genetics (Evolutionary Biology program), Uppsala University, Uppsala, Sweden; Ecology Division, Department of Zoology, Stockholm University, Stockholm, Sweden; Department of Cell and Molecular Biology, Molecular Evolution, Uppsala University, Uppsala, Sweden; Uppsala Multidisciplinary Center for Advanced Computational Science, Uppsala University, Uppsala, Sweden; National Genomics Infrastructure, Uppsala Genome Center, SciLifeLab, BioMedical Centre, Uppsala University, Uppsala, Sweden; Department of Ecology and Genetics (Evolutionary Biology program), Uppsala University, Uppsala, Sweden

**Keywords:** Y chromosome polymorphism, sex chromosome, sexual dimorphism, copy number variation, CNV, PacBio

## Abstract

The Y chromosome is theorized to facilitate evolution of sexual dimorphism by accumulating sexually antagonistic loci, but empirical support is scarce. Due to the lack of recombination, Y chromosomes are prone to degenerative processes, which poses a constraint on their adaptive potential. Yet, in the seed beetle, *Callosobruchus maculatus* segregating Y linked variation affects male body size and thereby sexual size dimorphism (SSD). Here, we assemble *C. maculatus* sex chromosome sequences and identify molecular differences associated with Y-linked SSD variation. The assembled Y chromosome is largely euchromatic and contains over 400 genes, many of which are ampliconic with a mixed autosomal and X chromosome ancestry. Functional annotation suggests that the Y chromosome plays important roles in males beyond primary reproductive functions. Crucially, we find that, besides an autosomal copy of the gene *target of rapamycin* (*TOR*), males carry an additional *TOR* copy on the Y chromosome. TOR is a conserved regulator of growth across taxa, and our results suggest that a Y-linked *TOR* provides a male specific opportunity to alter body size. A comparison of Y haplotypes associated with male size difference uncovers a copy number variation for *TOR*, where the haplotype associated with decreased male size, and thereby increased sexual dimorphism, has two additional *TOR* copies. This suggests that sexual conflict over growth has been mitigated by autosome to Y translocation of *TOR* followed by gene duplications. Our results reveal that despite of suppressed recombination, the Y chromosome can harbor adaptive potential as a male-limited supergene.

## Introduction

Driven by differences in reproductive strategies (Parker 2006; Schärer et al. 2012), males and females commonly experience sexually antagonistic selection on traits present in both sexes, for example (Andersson 1994; Arnqvist and Rowe 2005). When the sexes share genetic variation in homologous traits (Lande 1980), an allele beneficial to females may be deleterious for males and vice versa. This genetic dependency can hinder sex-specific adaptations, causing intralocus sexual conflict (Bonduriansky and Chenoweth 2009). The Y chromosome is limited to males, and linkage of sexually antagonistic loci on the Y would represent a straightforward solution to this genetic conflict (Charlesworth and Charlesworth 1980; Rice 1984; Charlesworth 2002; Mank 2012). A male limited pathway to alter the expression of a sexually antagonistic trait disconnects the genetic basis between the sexes and can reduce gender load by enabling not only males but also females to reach their fitness optima. Yet, the role of Y chromosomes in the evolution of sexual dimorphism has historically been neglected (Mank 2012). This is because the Y typically degenerates quickly after the loss of recombination with the X, which is expected to constrain its adaptive potential (Bachtrog 2013).

The Y chromosome degenerates due to reduced efficacy of selection and mutation accumulation (reviewed in Charlesworth and Charlesworth 2000), selective haploidization driven by regulatory evolution (Lenormand et al. 2020), or a combination thereof. As a consequence of degeneration, Y chromosomes are often heteromorphic, void of genes, and rich in repetitive elements. Y chromosome sequences are therefore challenging to assemble, and Y assemblies considered as complete are available only in a handful of mammalian species (Skaletsky et al. 2003; Hughes et al. 2010; Hughes et al. 2012; Hughes et al. 2020). There are also a few examples of near complete assemblies such as for malaria mosquitos (Hall et al. 2016), some fish (Peichel et al. 2020; Sandkam et al. 2021) and neo-Y in *Drosophila miranda* (Mahajan et al. 2018), and the affordability of high quality long read sequencing is expected to extend this list at a swift pace. Genes remaining on degenerate Y chromosomes are frequently translocated from the autosomes, allowing their male specialization. Y linked genes show typically testis specific expression, for example (Skaletsky et al. 2003; Hughes et al. 2012; Soh et al. 2014; Peichel et al. 2020), which suggests they are associated with male primary reproductive functions. But recently, heteromorphic Y chromosomes have also been shown to affect sexually dimorphic, nonreproductive traits in humans (Prokop and Deschepper 2015), drosophila (Nielsen et al. 2023), fish (Lampert et al. 2010; Sandkam et al. 2021; Singh et al. 2023), and seed beetles (Kaufmann et al. 2021), suggesting a wider role in nonsexual traits than previously appreciated (Cīrulis et al. 2022).

The order of Coleoptera (beetles) is the most species rich group of animals on the earth. Yet, only a handful of beetle species have been sequenced thus far (McKenna et al. 2019), and their Y chromosomes remain largely uncharted. Coleoptera has XY sex determination, but the sex determining genes involved are largely unknown. A study of karyotypes of over 4,000 beetle species shows occasional loss of Y chromosomes (Blackmon and Demuth 2014), suggesting that Y is not essential for sex determination (Blackmon et al. 2017). Interestingly, most studied species in the largest Coleopteran suborder Polyphaga do not have an obligate XY chiasmata formation and therefore lack a pseudo autosomal region (PAR) and XY recombination altogether (Blackmon and Demuth 2014). One such species is a seed beetle *Callosobruchus maculatus* that harbors a dot-like heteromorphic Y chromosome estimated to represent <2% of the genome (Sayadi et al. 2019). Based on cytogenetic data, the *C. maculatus* Y is euchromatic despite its small size (Angus et al. 2010).

We recently discovered segregating Y-linked genetic variance for male body size (Kaufmann et al. 2021). Female and male *C. maculatus* have different fitness optima for body size (Berg and Maklakov 2012; Berger et al. 2014a; Berger et al. 2016), which is closely connected to many other life history traits (Arnqvist et al. 2022). A combination of quantitative genetic analysis, artificial selection, and isolating the effect of Y linked genetic variance by introgressing the putative Y haplotypes onto a common genetic background revealed two phenotypically distinct male morphs associated with different Y haplotypes (Y_L_ and Y_S_ for large and small size, respectively). We demonstrated that selection on males can deplete Y haplotype variation quickly (Kaufmann et al. 2023) and that carrying either one of the Y haplotype alters the male size (weight), and consequently the level sexual size dimorphism, by 30% (Kaufmann et al. 2021). Body size is a classic quantitative trait, and although quantitative genetic evidence suggests that even the autosomal genetic architecture of body size in *C. maculatus* consist of a combination of few major effect loci and many small effect loci (Kaufmann et al. 2023), finding that a substantial part of its architecture in males is controlled by a nonrecombining “supergene” is surprising. But, a Y-linked element has also been implicated in male body size variation in humans (Ellis et al. 2001) and fish (Lampert et al. 2010; Sandkam et al. 2021; Singh et al. 2023) suggesting that Y-linkage may be a common way to mitigate sexual conflict over growth across diverse taxa.

In this study, we assembled previously uncharacterized X and Y chromosome sequences of *C. maculatus* and identified molecular differences between the two Y haplotypes (Y_S_ and Y_L_) associated with small or large male body size and with major effects on sexual size dimorphism, shedding light on the underlying molecular mechanisms. To do this, we took advantage of comparing genomes of the Y_S_ and Y_L_ introgression lines (Kaufmann et al. 2021) (hereafter referred to as S and L, respectively) that share inbred autosomal and X chromosomes and only differ in their respective Y haplotype. Here, we first identified nonrecombining X and Y contigs in both S and L genomes, by comparing sequence coverage difference between males and females, and verified male specificity of the longest Y contig (8.4Mb) with polymerase chain reaction (PCR). To further confirm the identity of the Y contigs and characterize Y variation, we compared the genomes of S and L lines that share variants in all other chromosomes except on the Y. We identified and functionally characterized protein coding genes and repeat structure of the Y contigs, analyzed the origins of the Y genes as X gametologs or autosomal paralogs, studied gene duplications within the Y to identify putative ampliconic genes, and examined expression of Y-linked genes.

## Results

### Genome Assembly

First, we separately assembled the genomes of the S and L lines and annotated the slightly more continuous S genome (carrying the ancestrally more frequent Y_S_ haplotype), which was subsequently used as the reference genome in this study. Assembly quality assessment using k-mer spectrum showed that only 1.5% of unique k-mers (containing repeats and low coverage reads) could not be assembled, whereas the k-mer completeness of the assembly (i.e., of primary and alternative) is >97%. In accordance, 99.9% of the hi-fi reads mapped back to the assembly with relatively even coverage. The assembled S genome has 938 contigs that yield a total genome length of 1.246 Gb, in close accordance with the genome size estimate of 1.2Gb for this species (Arnqvist et al. 2015). The N50 value of the assembly is 9.45 Mb with the longest contig being 37.4 Mb in length, the L50 is 38, and the BUSCO completeness scores over 98% (insecta_odb10: complete: 98.1% [single copy: 87.6%, double copy: 10.5%], fragmented: 0.2%, missing: 1.7%, *n* = 1367), demonstrating that the assembly is of a very high quality and further improves the previously published genome for this species (*C. maculatus* reference genome; N50 of 0.15 Mb and total genome size of 1.01 Gb Sayadi et al. 2019). Five hundred ninety-seven contigs were shorter than 100 kb (consisting of 24.9 Mb length, 2.00% of the total S assembly length) and were not considered in the downstream sex chromosome identification analysis because their chromosome type (autosome, X or Y) could not be determined with confidence due to their short length. The L line assembly is also of high quality with 1,323 contigs and a total length of 1.224 Gb, the longest contig being 30.2 Mb in length, with an N50 of 9.86 Mb, L50 of 38 and a BUSCO completeness score of over 97% (insecta_odb10: complete: 97.8% [single copy: 87.5%, double copy: 10.3%], fragmented: 0.3%, missing: 1.9%, *n* = 1367). Nine hundred forty-five contigs were shorter than 100 kb (consisting of 41.0 Mb, 3.35% of the total L assembly length).

After repeat-masking, 72.1% of the reference genome was soft-masked, of which 21.1% was identified as retroelements (primarily LINEs: 17.8%) and 21.0% as DNA transposons. A total of 24.8% interspersed repeats remained unclassified. Various low-complexity repeats formed the remainder of the soft-masked content. The final set of annotated gene models include 35,865 genes (68% increase compared with the original assembly Sayadi et al. 2019) and 39,983 transcripts (3,451 two-transcript gene models and 297 with more than two transcripts; 14% increase in the total number of transcripts Sayadi et al. 2019). A total of 25,651 transcripts received functional annotation.

Given our strict laboratory protocol, we did not expect any bacterial contaminations. Upon inspection, we did however find 16S genes from Enterococcus spp. in both L and S samples, and the same strains were present in both. The coverage for these contigs is five to seven times higher than the average coverage for genomic contigs and therefore way above the reference level for the initial detection of candidate sex-linked contigs. Importantly, our identification of Y linked contigs relies on several methods described below and is also guided by RNA-seq data from poly-A selected RNA, thus eliminating a risk of contaminating bacterial reads among the contigs identified as Y linked.

### Identification of Y and X Contigs

We performed a coverage comparison analysis (with sex assignment through coverage [SATC] Nursyifa et al. 2022) using Illumina short-read sequencing data from samples of both sexes (Sayadi et al. 2019) to identify novel sex chromosome sequences. SATC correctly identified the sexes of the samples. In the S genome assembly, four Y (total of 10.1 Mb) and eight X contigs (total of 58.6 Mb) were detected based on significant coverage differences, whereas in the slightly more fragmented L genome, we identified five Y contigs (total of 4.89 Mb) and ten X contigs (total of 64.2 Mb) (supplementary tables S1 and S2, Supplementary Material online). Importantly, the identified Y contigs from both assemblies map to each other, demonstrating that SATC identified homologous sequences in both assemblies (supplementary figs. S1 and S2, Supplementary Material online). The same contigs were identified whether using unfiltered data or when using repeat masked contigs, with minor exceptions (see supplementary tables S1 and S2 and figs. S1 and S2, Supplementary Material online). Note that the SATC analysis identified several additional contigs to show significant coverage difference between the sexes (see supplementary table S1, Supplementary Material online); however, the relative coverage difference in these cases was below 10%, and we therefore took a more conservative approach and only considered sex chromosome contigs above this threshold in our downstream analysis. It is possible however that these (or other unidentified) contigs are still sex linked but less diverged between X and Y.

**Fig. 1.**
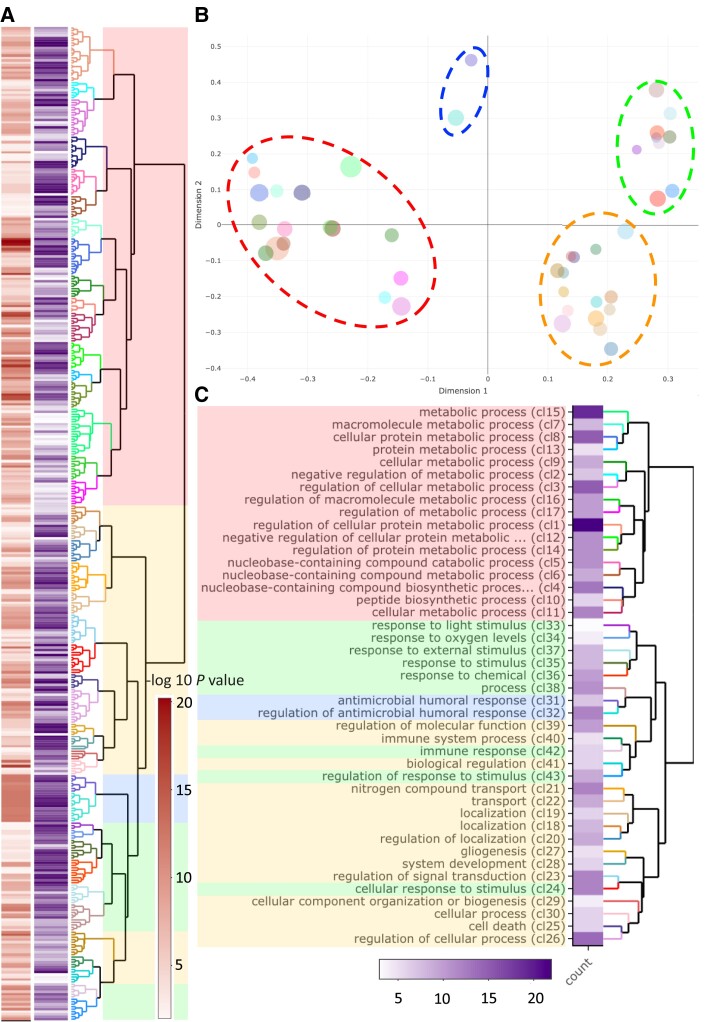
GO enrichment for Y-linked genes with “biological process” annotations. (*A*) Dendrogram of GO terms based on Wang's semantic similarity distance, heatmap (red) indicates statistical significance as -log_10_*P*-values (i.e., higher -log_10_*P*-values have higher statistical support) and information content (purple). (*B*) Multidimensional scaling (MDS) plot based on BMA distance, representing the proximities of dendrogram clusters in (*A*). Dot size indicates the number of GO terms within each cluster. We highlight the four major functional groups with dashed ellipsoids (red ≅ metabolic processes; blue ≅ antimicrobial response; green ≅ response to stimulus; yellow ≅ development, cell organization, growth, and cell apoptosis) (*C*) Dendrogram representation of clusters from (*B*) with GO term description of the first common GO ancestor and heatmap for the number of GO terms within each cluster.

Gene ontology (GO) enrichment of Y linked transcripts, as compared with all identified sex-linked transcripts, shows that Y is functionally different from the X (fig. 1). The significantly enriched processes on the Y include cell proliferation, regulation of development, cell death and apoptosis, response to stress/external stimulus such as response to starvation, immune response, RNA processing and regulation of posttranscriptional gene expression as well as protein modification (ubiquitination), and various metabolic processes (fig. 1; supplementary table S2, Supplementary Material online). GO enrichment of Y transcripts for molecular function is presented in supplementary figure S7, Supplementary Material online.

The identified Y contigs, in either the Y_S_ or the Y_L_ haplotype, do not seem to show a higher number of repeats per length, nor a higher percentage of repeat content, compared with the X or autosomal contigs (supplementary figs. S4 and S5, Supplementary Material online). However, the composition of repeat content on the Y is somewhat unique, where repeats identified as DNA/Maverick and LINE/Penelope are overrepresented, as a proportion of all repeat elements, on the Y compared with the X or autosomal contigs (supplementary fig. S6, Supplementary Material online).

#### Gametologs, Paralogs, and Ampliconic Genes

**Fig. 2.**
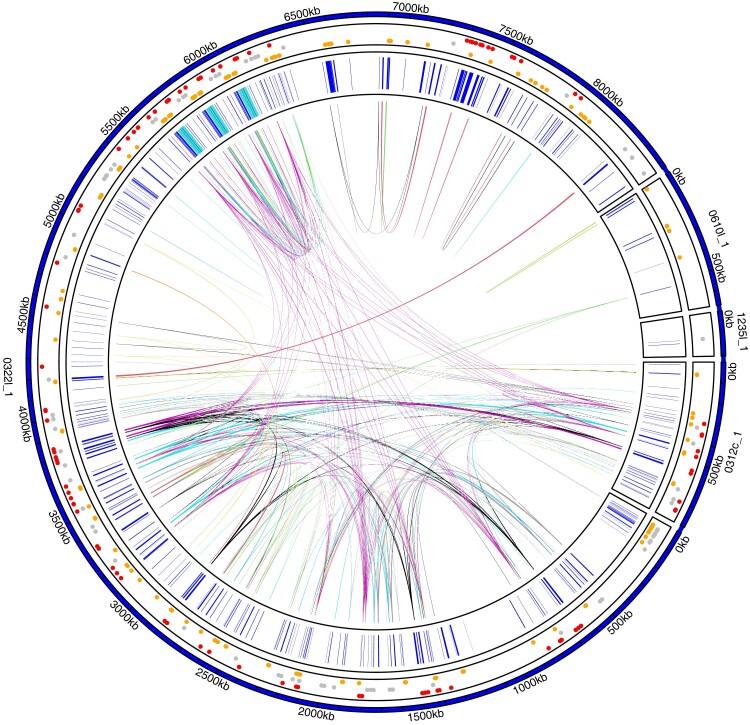
Overview of Y genes. Each amplicon group (i.e., genes with >99.9% nucleotide sequence identity and >95% query coverage) is highlighted with a genomic link. Two hundred nineteen out of a total of 437 Y genes have at least one additional copy on Y. Gene positions of all Y-linked genes are shown in inner track in blue; regions containing *TOR* are highlighted in turquoise (see fig. 4*A* for more details). The outer track indicates whether a gene has exclusively gametologs on the X (red, *n* = 99), paralogs on the autosomes (yellow, *n* = 157), or both (gray, *n* = 73); dots are scattered by homolog type. See supplementary figure S9, Supplementary Material online for amplicon groups on the X.

We detected 437 transcripts on the Y_S_, of which 202 transcripts are ampliconic, and have between 1 and 13 additional nearly identical copies on the Y (>99.9% nucleotide similarity), forming 67 ampliconic groups. Hence, we identified 302 unique Y-linked transcripts, of which 235 are nonampliconic transcripts and 67 form ampliconic groups. 424 transcripts have at least one gametolog or paralog in the genome (when searching for Y protein sequences against all *C. maculatus* proteins, using blastp Camacho et al. 2009 (>50% query coverage) and filtering for >80% sequence identity and E-value threshold = 1e-20). With these criteria, we detected in total 281 unique autosomal paralogs, 214 unique X-linked gametologs and 359 unique Y-linked homologous transcripts (supplementary fig. S8, Supplementary Material online). When lowering the sequence similarity threshold, we find homologs even for the remaining 12 Y-linked transcripts, although the best hit protein sequence similarity drops to below 40% for some of them. To identify autosomal or X ancestry for each Y transcript, we categorized them as exclusively autosomal paralogs (*n* = 157), exclusively gametologs on the X (*n* = 99) or homologs on both (*n* = 73) (fig. 2), while excluding genes that have homologs on uncategorized contigs (<100 kb, *n* = 51 genes). Y shared significantly more exclusive X gametologs than exclusive autosomal paralogs, when accounting for size difference (two-tailed Fisher's exact test: 95%CI (9.56, 16.07), *P* < 0.0001) or difference in the overall number of transcripts (two-tailed Fisher's exact test: 95% CI (8.32, 14.13), *P* < 0.0001). Gametologs that have been maintained on the Y are functionally enriched for RNA processing, particularly genes involved in RNA splicing, development as well as metabolic processes. Y transcripts with paralogs on the autosomes are functionally enriched for response to stimulus, developmental processes, protein translation and posttranslation modification (supplementary fig. S10, Supplementary Material online). Interestingly, this elevated sequence similarity between the X and the Y is not reflected in pronounced sequence synteny blocks between X and Y contigs, neither in nucleotide sequence nor gametolog synteny (supplementary fig. S11, Supplementary Material online).

### Characterization of the Y Variation Associated with the Body Size Difference

#### Variant Calling

The patterns of shared single nucleotide polymorphisms (SNPs) and fixed single nucleotide variants (SNVs) in the S and L genomes are well aligned with the expectations considering how the lines were created and further confirm the identity of the detected X and Y sequences. The majority of SNVs (2,823,154) are shared polymorphisms in both genomes (2,812,361 SNPs, 99.6%), and there are only few fixed SNV differences between the two introgression lines (10,793, 0.38%) (table 1). Autosomal contigs have significantly more shared SNP/bp than contigs identified as the sex chromosomes (two-tailed Fisher's exact test: 95% CI [28.8, 30.5], *P* < 0.0001% and 95% CI [83.2, 109.6], *P* < 0.0001, for the X and Y, respectively) (supplementary fig. S12, Supplementary Material online), and the X contigs have significantly more shared SNP/bp than the Y contigs (two-tailed Fisher's exact test: 95% CI (2.80, 3.71), *P* < 0.0001). In contrast, the Y contigs have significantly more fixed SNV differences/bp than autosomal contigs (two-tailed Fisher's exact test: 95%CI (43.4, 47.3), *P* < 0.0001), and there are no fixed SNV differences on the X contigs (supplementary fig. S13, Supplementary Material online).

**Table 1.** The Number of SNP and Fixed Differences between the Two Y Introgression Lines Split by Chromosome Type. Note that Contigs Shorter Than 100 kb Are Not Categorized as Y, X, or A.

	SNP	Fixed SNV	Total Length YS (bp)	Length Y_S_-Y_L_ Coverage [bp]
**A**	2,802,495	7,737	1,153,104,983	1,143,254,074
**X**	4,806	0	58,557,095	58,489,645
**Y**	212	2,622	10,112,272	8,276,151
**<100** **kb**	4,571	185	24,939,325	22,280,823

**Fig. 3.**
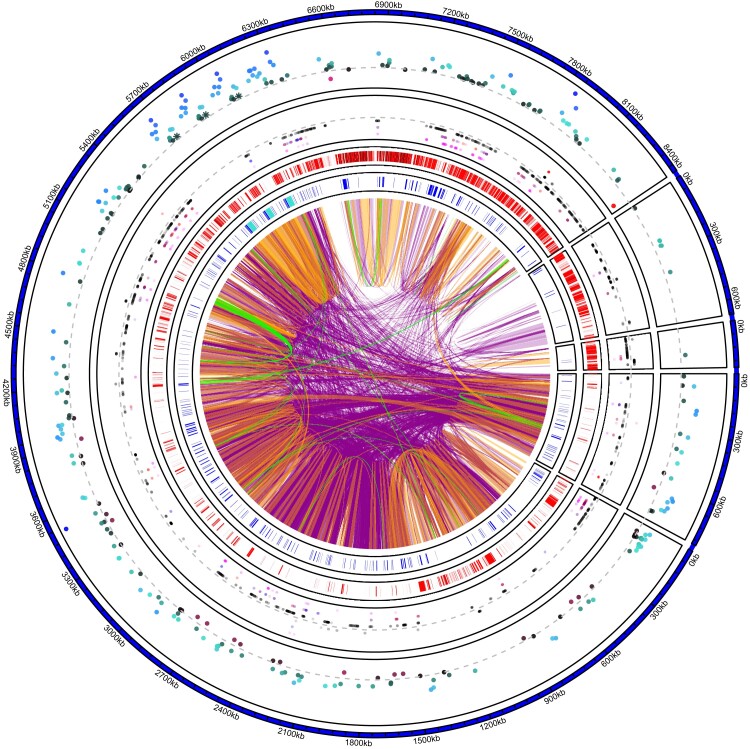
Overview of Y contigs in the Y_S_ genome. Color-coded genomic links show high level of nucleotide sequence similarity among and within Y contigs (purple > 99%, orange > 99.9%, green >99.99% nucleotide similarity matches > 1 kb in size). The inner track shows the position of annotated genes in blue, with the *TOR* regions highlighted in turquoise (fig. 4). The second inner track highlights areas of low mapping coverage between the two Y haplotypes: areas shown in red indicate coverage lower than 17× (i.e., regions that may lack coverage for reliable variant calling via DeepVariant). The third track shows the position of SNVs, SNPs (outside of the dashed gray line) and fixed SNV (inside the dashed gray line). SNVs in black (closest to the dashed gray line) are outside of gene regions; SNVs in magenta (farthest from the dashed gray line) are within gene regions. SNVs in blue and red are in 2 kb upstream or 2 kb downstream (proxy for cis-regulatory region of a gene) of a gene. The outer track shows differential gene expression between males and females. Genes in blue toward the outside are male biased; genes in red toward the center are female biased (gray dashed line is shown as a reference to indicate no difference in gene expression). *TOR* gene expressions are highlighted as asterisks and are significantly male biased. The Y_L_ haplotype is similarly repetitive as the Y_S_ haplotype shown here (see supplementary fig. S14, Supplementary Material online).

We identified a total of 137 genes with fixed SNV differences within the gene region or in close proximity to genes (i.e., ±2 kb as an approximation of cis-regulatory up and downstream area) between the two Y-haplotypes (fig. 3). For 12 of these 137 genes, we could identify unique *D. melanogaster* orthologs and annotate their function via FlyBase (Larkin et al. 2021) including DNA/RNA binding, mRNA splicing, regulation of cell proliferation and protein ubiquitination (supplementary table S3, Supplementary Material online).

#### Y-Linked Target of Rapamycin Amplicon

One of the annotated Y-linked genes indicated strong homology to the gene *target of rapamycin (TOR),* a highly conserved growth regulatory gene forming the IIS/TOR pathway (Wullschleger et al. 2006). Mapping a consensus *TOR* protein to our assembly via exonerate (Slater and Birney 2005) identified one autosomal *TOR* gene, detected in both Y_S_ and Y_L_ genomes (see supplementary methods, Supplementary Material online “genome annotation” for full details). In addition, there is one gene on the opposite strand that matches to adenosine deaminase 2 in several taxa (also involved in cell proliferation).

**Fig. 4.**
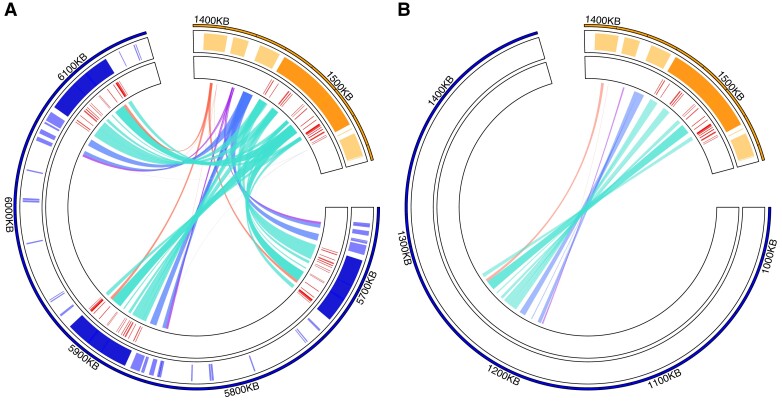
Zoom into the *TOR* region on the Y (blue) and on the autosome (yellow). The inner track shows the position of identified exons in red. Note that all Y linked *TOR* copies lack the exons 1–5 (from the 5′ end) and have one partial exon 6; all the other *TOR* exons (7–25) are present and complete. (*A*) Y_S_ haplotype, showing the three *TOR* regions (denoted with ^[a]^, ^[b]^, and ^[c]^). Note that we here only present the relevant region of the whole utg0003221_l Y_S_ contig (blue, see highlighted region in figs. 2 and 3). (*B*) Y_L_ contig containing the *TOR* region in the Y_L_ haplotype (blue, showing the whole contig) and the autosomal *TOR* region (yellow, from the annotated Y_S_ assembly). In the Y_L_ haplotype, we identify only one Y-linked copy.

We further discovered three consecutive copies of the Y *TOR* on the Y_S_ haplotype that causes the small body size morph in males (fig. 4*A*), but there is only a single Y-linked *TOR* in the Y_L_ haplotype associated with the large body size morph in males (fig. 4*B*), revealing Y-linked copy number variation (CNV) of the *TOR* region. Aligning Y_S_ and Y_L_ contigs (supplementary fig. S14, Supplementary Material online) shows that the *TOR* region CNV is located in the middle of an otherwise continuous alignment between two haplotypes, showing that CNV is not an artefact caused by a broken Y_L_ haplotype contig.

**Fig. 5.**
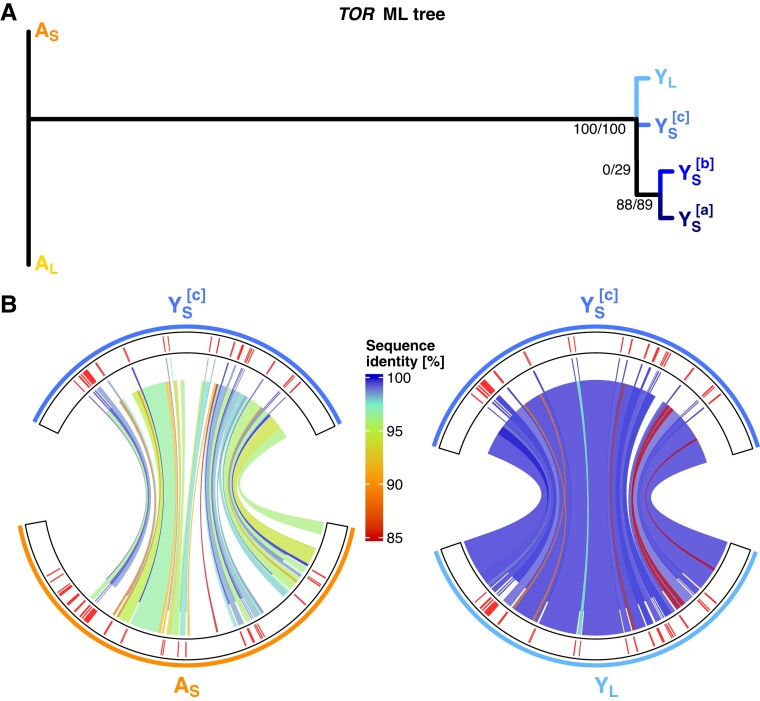
*TOR* region genealogy and comparison. (*A*) Maximum likelihood gene tree of exonic *TOR* sequences. Note that the A_S_ and A_L_ (highlighted in orange shades) have identical exonic sequences. High bootstrap values (100/100) indicate that Y-linked *TOR* (highlighted in blue shades) are more similar to each other than to the autosomal *TOR* and that Y_S_^[a]^ and Y_S_^[b]^ are sister sequences to each other (88/89). However, the clustering of the remaining Y-linked *TOR* sequences has low support (0/29). (*B*) Nucleotide alignment with Mummer of exonic and noncoding regions separately. The outer track shows the *TOR* exon positions in red. Genomic links show nucleotide similarity, exonic links are shifted upwards, whereas noncoding alignments are shifted down to guide easier distinction between the two regions. Fully factorial pairwise comparison of all *TOR* regions is presented in supplementary figure S15, Supplementary Material online. Left side: Comparison of the autosomal and the Y_S_^[c]^*TOR* region. Although there are structural differences (gaps) and low sequence identity matches in the noncoding regions, the exonic regions seem conserved between the autosomal and the Y_S_*TOR* copy. Note that all Y *TOR* regions lack the exon 1–5 (from the 5′ end) and exon 6 is only partially present. Exons 7–25 are present in all Y *TOR* regions. Right side: Comparison of Y_S_^[c]^*TOR* and the *TOR* region on the other Y haplotype (Y_L_). We find high nucleotide similarity in both noncoding and exonic regions alike, with one structural difference in a noncoding region.

A closer comparison of the autosomal and the Y linked *TOR* region reveals that all Y-linked *TOR* copies (in both Y_S_ and Y_L_ haplotypes) lack the initial five 5′ coding sequences (CDSs) compared with the autosomal *TOR*. A maximum likelihood tree (fig. 5*A*) of concatenated CDS comparing all *TOR* regions (i.e., the autosomal *TOR* of each assembly [A_S_ and A_L_], three Y-linked Y_S_*TOR* copies, and one Y-linked Y_L_*TOR*) shows the following: First, the autosomal A_S_ and A_L_*TOR* regions are isogenic, as expected. Second, all Y-linked *TOR* sequences cluster together with high bootstrap confidence, indicating that the *TOR* transposition from the autosome to the Y predates the two Y haplotypes. Also, DeepVariant SNP calling did not detect any SNV differences within the *TOR* region between the Y_S_ and Y_L_ haplotypes, which may also be due to lower coverage when mapping the two Y haplotypes against each other (fig. 3). We find with high confidence that Y_S_^[a]^ and Y_S_^[b]^*TOR* copies are most similar to each other but the remaining clustering of Y-linked *TOR* sequences has low support. Importantly, although the exons align with high similarity across all *TOR* regions, non-CDSs have diverged more and show lower sequence similarities (fig. 5*B*). This is particularly apparent when comparing autosomal and Y-linked *TOR* regions, where non-CDSs frequently do not align, indicating structural differences between them (fig. 5*B*; supplementary fig. S14, Supplementary Material online).

In addition to the CNV of the *TOR* locus, aligning the Y_S_ and Y_L_ haplotypes to each other suggests that there are further structural differences between the haplotypes (supplementary fig. S15, Supplementary Material online). Moreover, there are similarities between the classes and distribution of repetitive elements between the autosomal and the different copies of the Y-linked *TOR* region, indicating homology also at the level of *TOR*-associated repetitive elements (supplementary fig. S16, Supplementary Material online).

## Discussion

Here, we assembled two new *C. maculatus* genomes, and successfully identified 10 Mb of the Y chromosome, using a set of complementary methods, and analyzed the gene content of the Y sequences. We discovered that despite having lost most of its sequence since divergence from the X, the *C. maculatus* Y is rich in genes, many of which are expressed, with diverse functions from metabolism, development, and stimulus response to regulation of gene expression and translation. This is in line with cytogenetic data suggesting that the Y is mostly euchromatic (Angus et al. 2010), in spite of not recombining. We characterized molecular differences between the two Y haplotypes that were previously inferred from patterns of male limited inheritance of body size (Kaufmann et al. 2021). In addition to fixed single nucleotide differences in proximity of over a hundred genes, we identified a Y-linked copy of a functionally conserved and well-studied autosomal growth factor gene *TOR*, highlighting the potential for male specific growth regulation via the Y chromosome. Furthermore, we detected CNV of *TOR* between the two Y haplotypes underlying body size variation. Together our results indicate a central role of the Y chromosome in the evolution of sexual dimorphism in *C. maculatus* via male-specific evolution of *TOR*.

### XY Identification

Degenerate sex chromosomes are notoriously difficult to assemble (Rhie et al. 2022), and in *C. maculatus*, the entire genome has a high repeat content (Sayadi et al. 2019). Despite these challenges, long read sequencing yielded high quality and contiguous genome assemblies for both Y introgression lines that allowed us to identify large parts of both the X and the Y chromosomes, greatly extending and curating previously identified sex chromosome-linked portions of the genome. In *C. maculatus*, the size of the Y and X chromosomes has been estimated as ∼18 and 93Mb, respectively (Sayadi et al. 2019), based on a combination of flow cytometry and cytogenetic data, although the size estimate for the Y is uncertain (Arnqvist et al. 2015). We could assemble four Y contigs yielding 10.1 Mb, ∼56% of the current length estimate. By comparison, in *D. melanogaster* ∼10% of the Y has been assembled thus far (Hoskins et al. 2015), and to our knowledge, no Y assemblies exist yet for other beetle species. Additionally, we assembled a total of 58.6 Mb of the X chromosome, corresponding to approximately 63% of the estimated size. Sex chromosome contig identification depends on the quality and length of contigs, accuracy and completeness of the genome, and, importantly, on the state of differentiation between the X and Y chromosomes. Here, we rely on a suite of complementary methods including coverage difference between male and female reads, as well as genomic patterns of SNVs between our Y introgression lines that share the genome apart from the nonrecombining Y chromosome. Mapping of previously described putative X and Y contigs (Sayadi et al. 2019) also colocalize to our identified X and Y contigs in our assemblies, as expected. Furthermore, transcript expression patterns across the identified contig groups are in line with the unequal distribution of sex chromosomes between the sexes and show exclusive expression, or significantly higher male bias, of Y linked transcripts, and significantly less male bias for X-linked transcripts, as compared with the autosomal background. A large proportion of Y-linked transcripts show high sequence similarly with X-linked transcripts, in line with their common ancestry. As the expression data used here come from inbred lines that originate from the same ancestral population as the Y introgression lines, future work including gene expression data directly from the Y lines will provide a more complete view of Y linked gene expression.

Crucially, we confirmed the largest identified Y contig (8.4Mb)—carrying the identified Y linked *TOR* region—via male limited PCR amplification. The designed Y-specific primers work for both identified Y haplotypes and enables molecular sexing, a method that has thus far been lacking for *C. maculatus*.

### Gene Content on the Y

We find 437 Y-linked transcripts (417 genes) and the identified Y contigs show high gene density. The number of genes is similar to the gene rich Y chromosomes characterized in mouse (Soh et al. 2014) and bull (Hughes et al. 2020) but in contrast with human Y and other old insect Y chromosomes (*Drosophila*Zhang et al. 2020 and mosquito Hall et al. 2016) that harbor only few genes, which have been instrumental in forming the general expectation that Y-chromosomes are low in gene content (Mank 2012). Genes retained on the Y, despite its degeneration, can broadly be categorized into two classes: genes that are dosage sensitive X gametologs (Bellott et al. 2014; Bellott et al. 2017) and genes that are male beneficial, which may have been recruited to the Y via autosomal translocations. Many (often ampliconic) Y genes show testis limited expression and are likely central to the male reproductive function (Skaletsky et al. 2003; Murphy et al. 2006; Hughes et al. 2010; Soh et al. 2014; Janečka et al. 2018; Peichel et al. 2020). However, testis specific expression of highly amplified genes may also be indicative of meiotic drive (Soh et al. 2014; Hughes et al. 2020), in which case the involved genes need not be male beneficial but Y beneficial instead. Y is predicted to accumulate sexually antagonistic genes only beneficial to males (Rice 1984), but there is still little direct evidence to support Y-linkage of traits that are also present in females. Metabolic rate, body size, longevity (Berg and Maklakov 2012; Berger et al. 2014b; Berger et al. 2016), immunity, and gene expression (Immonen et al. 2017; Sayadi et al. 2019; Bagchi et al. 2021) are all sexually dimorphic traits in *C. maculatus*, indicating their evolutionary history under sexually antagonistic selection. We find functional enrichment of Y-linked genes that reflects these phenotypes remarkably well, including metabolic processes, immune response, response to stimulus and development, cell organization, growth, and cell apoptosis (fig. 1). This suggests that the Y-linked genes affect sexually dimorphic phenotypes beyond the body size (Kaufmann et al. 2021) and can offer a resolution to sexual conflict more broadly in *C. maculatus*.

Roughly 50% of the Y genes have either exclusively X gametologs (99 genes, 58.6 Mb) or autosomal paralogs (157 genes, 1,153 Mb) with >80% protein sequence similarity (fig. 2), indicating different origins for these genes. Notably, the number of identified X and autosomal homologs is positively correlated (Pearson correlation: t = 20.55, df 113, *P* < 0.001) for 115 Y-linked transcripts that have homologs on both regions (supplementary fig. S18, Supplementary Material online, note that this includes also transcripts that additionally have homologs on uncharacterized contigs), suggesting their coupling to transposable elements. The higher absolute number of genes acquired from the autosomes follows the pattern seen in *D. melanogaster* (Carvalho 2002) and humans (Skaletsky et al. 2003), where all or a substantial proportion of functional genes originate from autosomes, respectively. In contrast to known XY systems, in female heterogametic ZW taxa the W chromosomes mainly harbor Z gametologs, for example (Smeds et al. 2015). The difference is explained by sexual selection favoring transpositions to the Y in male heterogametic taxa (Carvalho 2002; Skaletsky et al. 2003; Hughes et al. 2012). The finding that *C. maculatus* Y contains a mix of male-specific but also seemingly functional X gametologs, with high sequence similarity retained to the X, suggests that the ancestral X genes have still important roles in males and may evolve under purifying selection. These X gametologs are enriched for functions related to development and metabolic processes as well as RNA processing/splicing (supplementary fig. S10, Supplementary Material online). Sex-specific RNA splicing allows expression of alternative transcripts in the sexes and can facilitate sexual dimorphism (Rideout et al. 2015; Rogers et al. 2021). However, whether the X gametologs are dosage-sensitive and sexually concordant or sexually antagonistic and on the Y specialized for male beneficial functions remain to be tested.

The male-specific Y genes acquired via transposition from autosomes are functionally enriched for a broader range of terms than the gametologs and account much of the general enrichment patterns observed across all Y genes (supplementary fig. S10, Supplementary Material online). An important avenue by which the Y chromosome can affect male phenotypes is by modulating gene expression throughout the genome, an effect described in *D. melanogaster* (Lemos et al. 2008) and for the *SRY* locus in mammals (Berta et al. 1990; Goodfellow and Lovell-Badge 1993). In line with such a mechanism, Y-autosome epistatic effects have also been associated with sexually antagonistic coloration in guppies (Paris et al. 2022). *Callosobruchus maculatus* Y shows significant enrichment for DNA binding molecular function, a category that *SRY* also falls into (supplementary fig. S7, Supplementary Material online), which is consistent with the idea that the Y chromosome has a wider regulatory role. The autosomal paralogs on the Y are enriched for genes involved in protein translation and posttranslational modification. Although transcriptional sex differences are the commonly evoked explanation for how sexual conflict can be resolved (Ellegren and Parsch 2007), this suggests that the Y chromosome may allow for translational modification to alter male beneficial phenotypic expression.

The Y contigs also contain a large number of sequences with strong homology to other Y loci, suggesting frequent gene duplication events, which is commonly observed in Y chromosomes (Skaletsky et al. 2003; Hughes et al. 2010; Soh et al. 2014; Brashear et al. 2018; Janečka et al. 2018; Ellison and Bachtrog 2019; Hughes et al. 2020; Peichel et al. 2020). The level of gene amplification we see is similar to the stickleback Y (Peichel et al. 2020) but less pronounced than in well-characterized mammalian Y chromosomes (Skaletsky et al. 2003; Hughes et al. 2010; Soh et al. 2014; Brashear et al. 2018; Janečka et al. 2018; Hughes et al. 2020). Amplification of genes on the Y may be fueled by sexual conflict over associated traits (Soh et al. 2014). Conservation of ampliconic genes is also observed in mammals (Brashear et al. 2018) and suggests that male specific amplified genes may have a large evolutionary advantage that withstand degenerative processes, such as Muller's ratchet (Brashear et al. 2018). Ampliconic genes tend to be expressed in the testis and enriched for male-specific reproductive functions in mammals (Skaletsky et al. 2003; Hughes et al. 2010; Soh et al. 2014; Janečka et al. 2018) and in stickleback (Peichel et al. 2020). But here, we could not yet detect any genes with obvious reproductive functions in males, based on GO terms or previously described *C. maculatus* seminal fluid proteins (*N* = 185) (Bayram et al. 2019).

X and Y do not form a chiasmata in *C. maculatus* and hence lack recombination by crossing-over and the PAR altogether. All species belonging to the *Callosobruchus* genus lack PAR based on their meiotic karyotypes (Blackmon and Demuth 2014), suggesting that XY divergence predates the genus. Long evolutionary history without recombination with the X could explain why our analysis of sequence similarity between X and Y does not indicate synteny between them (supplementary fig. S11, Supplementary Material online), nor do we find indications for inversion blocks that could have led to recombination suppression between X and Y. Ampliconic regions are prone to structural rearrangements (Jackson and Fink 1981; Skaletsky et al. 2003), which may cause further amplification and can also contribute to the lack of synteny between the X and Y.

### Molecular Differences Between the Y_S_ and Y_L_ Haplotypes Associated with Body Size Variation

We detected substantial molecular differences between the sequences of the Y_S_ and Y_L_ haplotypes associated with two distinct male limited body size morphs (Kaufmann et al. 2021). As expected for a nonrecombining, hemizygous genetic region, nearly all identified point mutation differences are fixed between the two Y haplotypes. The few detected SNPs could indicate real segregating polymorphisms (as the sample for sequencing consisted of multiple males) but are more likely artefacts caused by repetitive elements that add difficulty in genome assembly, accurate mapping, and variant calling. The 137 genes with fixed differences between the Y_S_ and Y_L_ haplotypes either in their coding or potential cis-regulatory regions are candidates to explain the phenotypic differences. Independent of body size, males with the Y_S_ haplotype also sire more offspring (*manuscript in preparation*). This could suggest that the Y haplotypes could cause differences in regulatory pathways associated with seminal fluid production or spermatogenesis, although we did not find any previously described *C. maculatus* seminal fluid proteins to be Y-linked. We identified *Drosophila* orthologs for 14 genes with fixed SNVs, and although we do not find any obvious causal candidate to explain the difference in reproductive capacity, we find two notable orthologs: *male-specific lethal* 2 and *ubiquitin specific protease 47*. *Male-specific lethal* 2 is a well-known regulator of dosage compensation between the X and Y in *D. melanogaster* (Zhou et al. 1995), which opens for differences in regulation of X-linked gene expression as a possible avenue to contribute to the phenotypic differences between the two Y haplotypes. Further, *ubiquitin specific protease 47* is known to interact with insulin/insulin-like signaling (IIS) pathway in *Drosophila* (Ashton-Beaucage et al. 2016), an important pathway that connects nutrient levels to metabolism, growth, development and longevity, which could affect the observed growth differences between the male morphs.

Remarkably, the longest *C. maculatus* Y chromosome contig also contains a *TOR* gene ortholog, the strongest candidate to explain the Y linked size variation between the sexes as well as in males. The TOR signaling pathway is highly conserved and present in organisms from bacteria and plants to animals and is one of the most ancient nutrient-sensing pathways (Wullschleger et al. 2006). The TOR pathway regulates growth and lifespan by coupling the growth factor signaling with nutrient sensing (Wullschleger et al. 2006). It is centrally involved in controlling cell metabolism, growth, proliferation and apoptosis. Together with IIS, TOR has also been implicated in differential gene expression between the sexes (Graze et al. 2018) and more specifically sexual size dimorphism in *D. melanogaster* (Rideout et al. 2015). To our knowledge it has never been detected on a sex chromosome before. But the potential for Y specific regulation of the TOR pathway has recently been implicated in the male polymorphic Poecilid fish *Poecilia parae*, where an inhibitor of the TOR pathway has been detected segregating on the Y (Sandkam et al. 2021). To understand whether the *C. maculatus TOR* gene on the Y represents ancestral homology with the X, or has occurred by a transposition from the autosomes after X-Y divergence, we searched the genome for any *TOR* copies. We could not detect the *TOR* on the X, but only in one of the autosomal contigs, supporting its origin on the Y by translocation. We detected multiple transposable element sequences flanking the *TOR* sequence in all of the Y copies as well as the autosomal one (supplementary fig. S17, Supplementary Material online), which could have played a role in the translocation and should be subject to further investigations.

Importantly, we detected *TOR* CNV between the Y_S_ and Y_L_ haplotypes, which makes this gene the most likely candidate for the striking difference in male body size between the two haplotypes, and thereby sexual size dimorphism (Kaufmann et al. 2021). The elevated intronic sequence divergence between the autosomal and Y-linked *TOR* copies suggests that the translocation of the *TOR* has occurred before the split of the two Y haplotypes, and also that sufficient time has passed since the translocation, to accumulate such differences in the introns. The exonic sequences have diverged only little, suggesting purifying selection on all copies to retain Y *TOR* regions as functional.

How the Y-linked *TOR* functions and putatively interacts with the autosomal *TOR* pathway presents important area for future research. The Y-linked, exonic *TOR* sequence is nearly identical to its autosomal paralog apart from five missing exons on the 5′end. The N-terminal of TOR proteins consist of HEAT repeats (Perry and Kleckner 2003) that mediate protein-protein interactions (Baretić et al. 2016). Empirical studies in *D. melanogaster* (Hennig and Neufeld 2002) and amoeba *Dictyostelium discoideum* (Swer et al. 2016) have demonstrated that overexpressing TOR inhibits cell growth and proliferation similar to loss of function mutants (Hennig and Neufeld 2002; Swer et al. 2016). Even a truncated extra copy of TOR is enough to reduce growth (Vilella-Bach et al. 1999; Hennig and Neufeld 2002). Gene copy number can correlate positively with gene expression (Shao et al. 2019), and *TOR* expression in adults in our data is overall male-biased (fig. 3). Paralog interference has been suggested as one possible consequence of gene duplications (Baker et al. 2013), whereby the paralogs can mutually exclude each other from binding with potential partners. The Y TOR could therefore affect male growth by interfering with the autosomal TOR pathway. The more common Y_S_ haplotype that makes males roughly 30% smaller than the Y_L_ haplotype has two additional copies of *TOR* on the Y, suggesting that the additional copies lead to further growth inhibition. Future work can establish how each of the copies in the two haplotypes may be expressed and function in regulating growth.

Recombination is a key mechanism generating and maintaining allelic diversity across loci. Finding substantial diversity in the absence of recombination is therefore unexpected, but echoes similar recent findings in Y-polymorphic fish species (Sandkam et al. 2021; Singh et al. 2023), including *Xiphophorus* where CNV in Y linked *melanocortin 4 receptor* gene is associated with male limited body size polymorphism (Lampert et al. 2010). Genetic variation should be rapidly fixed by selection and drift on the Y chromosome. Finding segregating Y polymorphism in both copy number and SNV differences in proximity to over 100 genes therefore suggests that processes such as frequency dependent selection likely have maintained these Y haplotypes in the population for a longer evolutionary time. Our identification of over 400 genes on the *C. maculatus* Y, despite the evidence of its degeneration, suggests that males can harbor substantial evolutionary potential through their Y chromosomes. Y translocation and *TOR* duplications reveal how evolutionary novelty in a conserved gene can be generated, adding to the growing literature that posit variation in gene copy numbers as central to generating genetic variance, and thereby to adaptive evolution.

## Materials and Methods

### Study Organism and Generation of the Y-Lines

As a model organism to study sexual conflict, much is known about sexual antagonism in the seed beetle *C. maculatus*. Aphagous adult *C. maculatus* females oviposit directly onto legume seed pods, within which larvae will develop for about 3 weeks, which allows for large scale experiments across multiple generations. The populations used in this study all stem from originally field caught (2010) individuals from Lomé, Togo (more details in Berger et al. 2014a) and have been kept in the lab for ∼200 generations as 41 isofemale lines.

The creation of Y_S_ and Y_L_ haplotype introgression lines is described in detail in (Kaufmann et al. 2021). Briefly, replicated bidirectional selection was applied on male body size for 10 generations, giving rise to L and S males. We then crossed S and L males from these selection lines with females from a single inbred line (originating from the same Lomé base population as the selection lines, inbred for >20 generations Grieshop and Arnqvist 2018), respectively. At each subsequent generation, sons were backcrossed to females from the maternal line, for a total of 11 generations, after which the lines were sequenced. The backcrossing scheme replaced the original autosomes and the X chromosome with those from the inbred line while maintaining the nonrecombining parts of the Y chromosome of the founding S or L males. Subsequent analysis of the body sizes confirmed the presence of two distinct male body size classes, whereas there was no difference in female body size (Kaufmann et al. 2021). We chose a single line representing each of the Y_S_ and Y_L_ haplotypes for sequencing.

The beetles used for the sequencing were reared under strictly standardized and controlled laboratory conditions, to minimize any environmental variation including risk of infestations. The larval food (mung beans, *Vigna radiata*, which is the only food source as adults do not eat) was stored in −20 °C for several months prior to use. The beetles were given ad libitum access to food, and reared under low density in incubators with 12:12 light, 29 °C temperature and 50% humidity.

### Sequencing and Genome Assembly

#### DNA Extraction and Library Preparation

For the extraction of high molecular weight (HMW) DNA, we flash froze adult virgin males (within 24 h after emergence) in liquid nitrogen. Individual abdomens were dissected on ice to avoid thawing of the tissue by removing head, thorax and the elytra. Five male abdomens were pooled and ground into fine powder with liquid nitrogen and a precooled pestle. The QIAGEN Genomic-Tip 20/g kit was used to extract HMW DNA, following the manufacturer's protocol, with an over-night incubation time for cell lysis (i.e., 12 h). To achieve the required amounts of HMW DNA, we pooled two samples (total of 10 males per Y introgression line). Five micrograms of genomic DNA were sheared on a Megaruptor3 instrument (Diagenode, Seraing, Belgium) to a fragment size of about 13–16 kb. The SMRTbell library was prepared according to Pacbio's Procedure & Checklist—Preparing HiFi Libraries from low DNA input using SMRTbell Express Template Prep Kit 2.0 (Pacific Biosciences, Menlo Park, CA, USA). The SMRTbells were sequenced on a Sequel IIe instrument, using the Sequel II sequencing plate 2.0, binding kit 2.2 on three Sequel II SMRT Cell 8 M per introgression line, with a movie time of 30 h and a pre-extension time of 2 h.

The genomes of the Y_S_ and the Y_L_ introgression lines were assembled individually using hifiasm (v. 0.7-dirty-r25) (Cheng et al. 2021) with default settings, yielding the S and L genomes. For each, the raw primary and alternative assemblies from hifiasm were subsequently separated into two approximately equal sized haplotype assemblies with purge_dups (v. 1.2.5, default parameters) (Guan et al. 2020). Following the purge_dup partitioning of repeats and low coverage contigs that could not be assembled, the k-mer completeness of the primary and alternative assemblies was checked again with Merqury, and the read mapping rate back to the final assembly assessed using default mapping parameters. Genome assembly completeness was also assessed via BUSCO (Manni et al. 2021), using default parameters on the insecta_odb10 database. We then chose the S genome (containing the ancestrally more frequently occurring Y_S_ haplotype) to be the reference genome, that we subsequently soft-masked for repetitive content (Arnqvist et al. 2023) using RepeatMasker (v. 4.1.2) (Smit et al. 2015) and fully annotated using the BRAKER (v. 2.1.6) (Hoff et al. 2019)/TSEBRA (v. 1.0.3) (Gabriel et al. 2021) pipeline. Detailed annotation methods are provided in the supplementary methods, Supplementary Material online “genome annotation.”

### Identification of Y and X Contigs

#### Mapping of Putative X and Y Contigs

We used gmap (v. 2021-03-08) (Wu and Watanabe 2005) default setting to quantify the number of hits of putative X and Y contigs, previously identified in *C. maculatus* (Sayadi et al. 2019), to our assembled contigs in both genomes. Mapping to contigs shorter than 100 kb was excluded.

#### Sex Assignment Through Coverage

To independently identify putative sex-linked contigs in each of our assemblies, we employed SATC (Nursyifa et al. 2022), which uses normalized coverage information across contigs to first identify XX and XY individuals from sets of male and female samples (Nursyifa et al. 2022). Informed by a *t*-test, SATC compares normalized coverage at each contig between XX and XY individuals to find contigs with significantly different coverage. At sex-linked chromosomes, specific XY:XX coverage ratios are expected for X-linked contigs (0.5:1) and Y-linked chromosomes (1:0) (Nursyifa et al. 2022). In practice, coverage can be highly variable resulting in deviations from the strict expectation. Here, we used the SATC approach to identify any contigs that had significantly different coverage between XX and XY individuals. We collected Illumina short-read sequencing data from Sayadi et al. (2019; ENA accession numbers: ERR3053159, ERR3053160, ERR3053163, ERR3053164, ERR3053161, ERR3053162, ERR3053165, and ERR3053166) (Sayadi et al. 2019). To increase certainty, we limited ourselves to the analysis of contigs >100 kb in length. Contigs that are shorter than this account for a total of 24′939′325 bp and make up only 2.00% of the reference genome (1′246′713′675 bp). For more details, see supplementary methods, Supplementary Material online “sex assignment through coverage (SATC).”

#### Polymerase Chain Reaction

We confirmed male-specificity of the longest identified Y contig with a multiplexed PCR, using two primers pairs, one that is Y specific and amplifies a 297 bp on utg000322l_1 product and a primer pair that amplifies an autosomal product of 189 bp length on utg000177l_1 as a positive control. For more details, see supplementary methods, Supplementary Material online “Molecular sexing.”

#### Gene Expression Analysis

To assess how the identified sex-linked genes may be expressed, we used gene expression data from virgin adult males (*n* = 29) and females (*n* = 32) (Kaufmann 2022). The expression data were collected from reproductive tissues of the abdomen of virgin individuals from different inbred lines that originate from the same Lomé base population as the Y lines. We used splice variant aware mapping of transcript via STAR (v. 2.7.2b) with default settings (Dobin et al. 2013). We then used picard (v. 2.23.4) (Broad Institute 2019) to mark duplicates and subread (v. 2.0.0) featurecount (Liao et al. 2014) to summarize exons by gene IDs allowing for multimappers due to high gene duplications on the Y. Additionally, we also summarized exons by gene IDs using default setting (i.e., no multimapping). We then used DESeq2 (Love et al. 2014) to analyze the gene expression patterns in males and females. We split the dataset into genes on the autosomes and the identified X and the Y contigs.

#### GO Enrichment on the Y

We examined how the Y chromosome is functionally diverged from the X using GO enrichment analysis. For this, we used the topGO R package (Alexa and Rahnenführer 2022), with nodeSize parameter of 10, comparing the frequency of terms among the Y linked transcripts to those among all transcripts on the sex chromosomes (X and Y). Visualization and clustering of the GO enrichment analysis was done using ViSEAGO R package (Brionne et al. 2019), clustering GO terms based on Wang's semantic similarity distance and ward.D2. Further aggregating of semantic similarity GO clusters was done with best-match average (BMA) method, as implemented in the ViSEAGO package.

#### Identification of Y Homologs on the X and the Autosomes

To identify whether the genes on the Y represent X gametologs or paralogs translocated from the autosomes, we used blastp (v.2.12.0) (Camacho et al. 2009) to compare Y proteins against all proteins in our reference assembly, requiring a minimum Y protein coverage of >50% and at least >80% AA identity, excluding self matches. To test whether X contigs show elevated number of Y gametologs due to shared XY ancestry, we compared the number of Y transcripts for which we exclusively find gametologs on the X, to the number of Y transcripts with exclusively paralogs on the autosomes, while accounting for difference in length or number of transcripts between autosomes and the X.

#### Y Amplicons

The mammalian Y chromosomes contain large ampliconic regions enriched with high-identity segmental duplications, for example (Skaletsky et al. 2003). Given that there seem to be a lot of duplicated genes also on the *C. maculatus* Y, we used blastn (v. 2.12.0) (Camacho et al. 2009) to blast all concatenated CDS for each transcript on the Y contigs against each other with stringent requirements of >95% query coverage and >99.9% sequence identity and excluding self matches, to detect amplicon groups on the Y.

### Characterization of the Y_S_ and Y_L_ Haplotypes

#### Variant Calling

We used minimap2 (v. 2.18-r1015) (Li 2018) to align reads to the reference allowing for up to 20% sequence divergence (asm20), as was recommended for HiFi reads. Duplicates have been marked with picard (v. 2.23.4) (Broad Institute 2019). We then used DeepVariant (v. 1.3.0, default settings) (Poplin et al. 2018) with model type PACBIO for HiFi reads to call variants and GLnexus (v. 1.4.1) (Lin et al. 2018) to merge the variant calling files for both Y haplotype lines. We used vcftools (Danecek et al. 2011) to filter the VCF files to only get SNVs with a minimum depth of 5 and an upper depth cutoff of 65. SNV were categorized into SNPs that are shared between both Y introgression lines (SNP) and single nucleotide differences that are fixed between the two Y-introgression lines (fixed SNV).

#### TOR Candidate Gene Analysis

A copy of a conserved gene coding for TOR was discovered in a putative Y contig identified in the study that sequenced *C. maculatus* genome for the first time (Sayadi et al. 2019). TOR is well known for its role in regulating growth across taxa (reviewed in Wullschleger et al. 2006) and is therefore a prime candidate to explain the male body size difference between the Y_S_ and Y_L_ haplotypes. We identified and curated TOR locations in the genome (see supplementary methods, Supplementary Material online “genome annotation” for full details). To compare the different TOR regions, we used MAFFT (v. 7.407, with –ep 0 –genafpair parameters) (Katoh et al. 2002) to align concatenated *TOR* CDS of each identified region in both assembled genomes and create a guided gene tree. To assess whether there are differences in exonic and non-CDSs divergence across the different *TOR* regions, we used fully factorial pairwise alignment of exonic and non-CDSs separately. For the noncoding alignment, we masked identified exonic regions with bedtools maskfasta and then aligned the non-CDSs with masked exons via Mummer nucmer (v. 4.0.0 with –maxmatch –c 100 parameters). For the exonic alignment, we used the same procedure but masking the noncoding regions instead. We visualized the exonic and noncoding alignments with the R package circlize (Gu et al. 2014).

## Supplementary Material

msad167_Supplementary_Data

## Data Availability

Sequencing data are available on ENA study_ID PRJEB62873, and the code is available in Zenodo (DOI: 10.5281/zenodo.7948013). ***Conflict interest statement.*** The authors declare no competing interests.

## References

[msad167-B1] Alexa A , Rahnenführer J. 2022. topGO: enrichment analysis for gene ontology. R Package Version 2.52.0.

[msad167-B2] Andersson M . 1994. Sexual seleciton. Princeton (NJ): Princeton University Press.

[msad167-B3] Angus RB , Dellow J, Winder C, Credland PF. 2010. Karyotype differences among four species of Callosobruchus Pic (Coleoptera: Bruchidae). J Stored Prod Res. 47:76–81.

[msad167-B4] Arnqvist G , Sayadi A, Immonen E, Hotzy C. 2023. A near chromosome-level assembly of the seed beetle Callosobruchus maculatus genome with annotation of its repetitive elements. *Submitt*. *MS*.

[msad167-B5] Arnqvist G , Rönn J, Watson C, Goenaga J, Immonen E. 2022. Concerted evolution of metabolic rate, economics of mating, ecology, and pace of life across seed beetles. Proc Natl Acad Sci U S A. 119:e2205564119. 10.1073/pnas.2205564119 PMC9388118 35943983

[msad167-B6] Arnqvist G , Rowe L. 2005. Sexual conflict. Princeton (NJ): Princeton University Press.

[msad167-B7] Arnqvist G , Sayadi A, Immonen E, Hotzy C, Rankin D, Tuda M, Hjelmen CE, Johnston JS. 2015. Genome size correlates with reproductive fitness in seed beetles. Proc Biol Sci. 282:20151421. 26354938 10.1098/rspb.2015.1421 PMC4614751

[msad167-B8] Ashton-Beaucage D , Lemieux C, Udell CM, Sahmi M, Rochette S, Therrien M. 2016. The deubiquitinase USP47 stabilizes MAPK by counteracting the function of the N-end rule ligase POE/UBR4 in Drosophila. PLoS Biol. 14:e1002539. 27552662 10.1371/journal.pbio.1002539 PMC4994957

[msad167-B9] Bachtrog D . 2013. Y chromosome evolution: emerging insights into processes of Y chromosome degeneration. Nat Rev Genet. 14:113–124. 23329112 10.1038/nrg3366 PMC4120474

[msad167-B10] Bagchi B , Corbel Q, Khan I, Payne E, Banerji D, Liljestrand-Rönn J, Martinossi-Allibert I, Baur J, Sayadi A, Immonen E, et al 2021. Sexual conflict drives micro- and macroevolution of sexual dimorphism in immunity. BMC Biol. 19:114. 34078377 10.1186/s12915-021-01049-6 PMC8170964

[msad167-B11] Baker CR , Hanson-Smith V, Johnson AD. 2013. Following gene duplication, paralog interference constrains transcriptional circuit evolution. Science 342:104–108. 24092741 10.1126/science.1240810 PMC3911913

[msad167-B12] Baretić D , Berndt A, Ohashi Y, Johnson CM, Williams RL. 2016. Tor forms a dimer through an N-terminal helical solenoid with a complex topology. Nat Commun. 7:11016. 27072897 10.1038/ncomms11016 PMC4833857

[msad167-B13] Bayram H , Sayadi A, Immonen E, Arnqvist G. 2019. Identification of novel ejaculate proteins in a seed beetle and division of labour across male accessory reproductive glands. Insect Biochem Mol Biol. 104:50–57. 30529580 10.1016/j.ibmb.2018.12.002

[msad167-B14] Bellott DW , Hughes JF, Skaletsky H, Brown LG, Pyntikova T, Cho T-J, Koutseva N, Zaghlul S, Graves T, Rock S, et al 2014. Mammalian Y chromosomes retain widely expressed dosage-sensitive regulators. Nature 508:494–499. 24759411 10.1038/nature13206 PMC4139287

[msad167-B15] Bellott DW , Skaletsky H, Cho T-J, Brown L, Locke D, Chen N, Galkina S, Pyntikova T, Koutseva N, Graves T, et al 2017. Avian W and mammalian Y chromosomes convergently retained dosage-sensitive regulators. Nat Genet. 49:387–394. 28135246 10.1038/ng.3778 PMC5359078

[msad167-B16] Berg EC , Maklakov AA. 2012. Sexes suffer from suboptimal lifespan because of genetic conflict in a seed beetle. Proc Biol Sci. 279:4296–4302. 22915670 10.1098/rspb.2012.1345 PMC3441075

[msad167-B17] Berger D , Berg EC, Widegren W, Arnqvist G, Maklakov AA. 2014a. Multivariate intralocus sexual conflict in seed beetles. Evolution 68:3457–3469. 25213393 10.1111/evo.12528

[msad167-B18] Berger D , Grieshop K, Lind MI, Goenaga J, Maklakov AA, Arnqvist G. 2014b. Intralocus sexual conflict and environmental stress. Evolution 68:2184–2196. 24766035 10.1111/evo.12439

[msad167-B19] Berger D , Martinossi-Allibert I, Grieshop K, Lind MI, Maklakov AA, Arnqvist G. 2016. Intralocus sexual conflict and the tragedy of the commons in seed beetles. Am Nat. 188:E98–E112. 27622882 10.1086/687963

[msad167-B20] Berta P , Hawkins JR, Sinclair AH, Taylor A, Griffiths BL, Goodfellow PN, Fellous M. 1990. Genetic evidence equating SRY and the testis-determining factor. Nature 348:448–450. 2247149 10.1038/348448A0

[msad167-B21] Blackmon H , Demuth JP. 2014. Estimating tempo and mode of Y chromosome turnover: explaining Y chromosome loss with the fragile Y hypothesis. Genetics 197:561–572. 24939995 10.1534/genetics.114.164269 PMC4063915

[msad167-B22] Blackmon H , Ross L, Bachtrog D. 2017. Sex determination, sex chromosomes, and karyotype evolution in insects. J Hered. 108:78–93. 27543823 10.1093/jhered/esw047 PMC6281344

[msad167-B23] Bonduriansky R , Chenoweth SF. 2009. Intralocus sexual conflict. Trends Ecol Evol. 24:280–288. 19307043 10.1016/j.tree.2008.12.005

[msad167-B24] Brashear WA , Raudsepp T, Murphy WJ. 2018. Evolutionary conservation of Y chromosome ampliconic gene families despite extensive structural variation. Genome Res. 28:1841–1851. 30381290 10.1101/gr.237586.118 PMC6280758

[msad167-B25] Brionne A , Juanchich A, Hennequet-Antier C. 2019. ViSEAGO: a Bioconductor package for clustering biological functions using gene ontology and semantic similarity. BioData Min. 12:16. 31406507 10.1186/s13040-019-0204-1 PMC6685253

[msad167-B26] Broad Institute . 2019. Picard Toolkit. GitHub Repository. Available from: http://broadinstitute.github.io/picard/.

[msad167-B27] Camacho C , Coulouris G, Avagyan V, Ma N, Papadopoulos J, Bealer K, Madden TL. 2009. BLAST+: architecture and applications. BMC Bioinformatics. 10:1–9. 20003500 10.1186/1471-2105-10-421 PMC2803857

[msad167-B28] Carvalho AB . 2002. Origin and evolution of the Drosophila Y chromosome. Curr Opin Genet Dev. 12:664–668. 12433579 10.1016/s0959-437x(02)00356-8

[msad167-B29] Charlesworth D . 2002. Plant sex determination and sex chromosomes. Heredity (Edinb). 88:94–101. 11932767 10.1038/sj.hdy.6800016

[msad167-B30] Charlesworth D , Charlesworth B. 1980. Sex differences in fitness and selection for centric fusions between sex-chromosomes and autosomes. Genet Res. 35:205–214. 6930353 10.1017/s0016672300014051

[msad167-B31] Charlesworth B , Charlesworth D. 2000. The degeneration of Y chromosomes. Philos Trans R Soc Lond B Biol Sci. 355:1563–1572. 11127901 10.1098/rstb.2000.0717 PMC1692900

[msad167-B32] Cheng H , Concepcion GT, Feng X, Zhang H, Li H. 2021. Haplotype-resolved de novo assembly using phased assembly graphs with hifiasm. Nat Methods. 18:170–175. 33526886 10.1038/s41592-020-01056-5 PMC7961889

[msad167-B33] Cīrulis A , Hansson B, Abbott JK. 2022. Sex-limited chromosomes and non-reproductive traits. BMC Biol. 20:1–18. 35794589 10.1186/s12915-022-01357-5 PMC9261002

[msad167-B34] Danecek P , Auton A, Abecasis G, Albers CA, Banks E, DePristo MA, Handsaker RE, Lunter G, Marth GT, Sherry ST, et al 2011. The variant call format and VCFtools. Bioinformatics 27:2156–2158. 21653522 10.1093/bioinformatics/btr330 PMC3137218

[msad167-B35] Dobin A , Davis CA, Schlesinger F, Drenkow J, Zaleski C, Jha S, Batut P, Chaisson M, Gingeras TR. 2013. STAR: ultrafast universal RNA-seq aligner. Bioinformatics 29:15–21. 23104886 10.1093/bioinformatics/bts635 PMC3530905

[msad167-B36] Ellegren H , Parsch J. 2007. The evolution of sex-biased genes and sex-biased gene expression. Nat Rev Genet. 8:689–698. 17680007 10.1038/nrg2167

[msad167-B37] Ellis JA , Stebbing M, Harrap SB. 2001. Significant population variation in adult male height associated with the Y chromosome and the aromatase gene. J Clin Endocrinol Metab. 86:4147–4150. 11549641 10.1210/jcem.86.9.7875

[msad167-B38] Ellison C , Bachtrog D. 2019. Recurrent gene co-amplification on Drosophila X and Y chromosomes. PLoS Genet. 15:e1008251. 31329593 10.1371/journal.pgen.1008251 PMC6690552

[msad167-B39] Gabriel L , Hoff KJ, Brůna T, Borodovsky M, Stanke M. 2021. TSEBRA: transcript selector for BRAKER. BMC Bioinformatics. 22:566. 34823473 10.1186/s12859-021-04482-0 PMC8620231

[msad167-B40] Goodfellow PN , Lovell-Badge R. 1993. SRY and sex determination in mammals. Annu Rev Genet. 27:71–92. 8122913 10.1146/annurev.ge.27.120193.000443

[msad167-B41] Graze RM , Tzeng RY, Howard TS, Arbeitman MN. 2018. Perturbation of IIS/TOR signaling alters the landscape of sex-differential gene expression in Drosophila. BMC Genomics. 19:893. 30526477 10.1186/s12864-018-5308-3 PMC6288939

[msad167-B42] Grieshop K , Arnqvist G. 2018. Sex-specific dominance reversal of genetic variation for fitness. PLoS Biol. 16:e20068101–21. 10.1371/journal.pbio.2006810 PMC6303075 30533008

[msad167-B43] Gu Z , Gu L, Eils R, Schlesner M, Brors B. 2014. Circlize implements and enhances circular visualization in R. Bioinformatics 30:2811–2812. 24930139 10.1093/bioinformatics/btu393

[msad167-B44] Guan D , McCarthy SA, Wood J, Howe K, Wang Y, Durbin R. 2020. Identifying and removing haplotypic duplication in primary genome assemblies. Bioinformatics 36:2896–2898. 31971576 10.1093/bioinformatics/btaa025 PMC7203741

[msad167-B45] Hall AB , Papathanos PA, Sharma A, Cheng C, Akbari OS, Assour L, Bergman NH, Cagnetti A, Crisanti A, Dottorini T, et al 2016. Radical remodeling of the Y chromosome in a recent radiation of malaria mosquitoes. Proc Natl Acad Sci U S A. 113:E2114–E2123. 27035980 10.1073/pnas.1525164113 PMC4839409

[msad167-B46] Hennig KM , Neufeld TP. 2002. Inhibition of cellular growth and proliferation by dTOR overexpression in Drosophila. Genesis 34:107–110. 12324961 10.1002/gene.10139

[msad167-B47] Hoff K , Lomsadze A, Borodovsky M, Stanke M. 2019. Whole-genome annotation with BREAKER. In: Kollmar M, editors. Gene prediction. Totowa (NJ): Humana Press. p. 65–95. doi:10.1007/978-1-4939-9173-0. PMC6635606 31020555

[msad167-B48] Hoskins RA , Carlson JW, Wan KH, Park S, Mendez I, Galle SE, Booth BW, Pfeiffer BD, George RA, Svirskas R, et al 2015. The release 6 reference sequence of the *Drosophila melanogaster* genome. Genome Res. 25:445–458. 25589440 10.1101/gr.185579.114 PMC4352887

[msad167-B49] Hughes JF , Skaletsky H, Brown LG, Pyntikova T, Graves T, Fulton RS, Dugan S, Ding Y, Buhay CJ, Kremitzki C, et al 2012. Strict evolutionary conservation followed rapid gene loss on human and rhesus y chromosomes. Nature 483:82–87. 22367542 10.1038/nature10843 PMC3292678

[msad167-B50] Hughes JF , Skaletsky H, Pyntikova T, Graves TA, van Daalen SK, Minx PJ, Fulton RS, McGrath SD, Locke DP, Friedman C, et al 2010. Chimpanzee and human y chromosomes are remarkably divergent in structure and gene content. Nature 463:536–539. 20072128 10.1038/nature08700 PMC3653425

[msad167-B51] Hughes JF , Skaletsky H, Pyntikova T, Koutseva N, Raudsepp T, Brown LG, Bellott DW, Cho T-J, Dugan-Rocha S, Khan Z, et al 2020. Sequence analysis in *Bos taurus* reveals pervasiveness of X–Y arms races in mammalian lineages. Genome Res. 31:1716–1726. 10.1101/gr.269902.120 PMC7706723 33208454

[msad167-B52] Immonen E , Sayadi A, Bayram H, Arnqvist G. 2017. Mating changes sexually dimorphic gene expression in the seed beetle *Callosobruchus maculatus*. Genome Biol Evol. 9:677–699. 28391318 10.1093/gbe/evx029 PMC5381559

[msad167-B53] Jackson JA , Fink GR. 1981. Gene conversion between duplicated genetic elements in yeast. Nature 292:306–311. 6265790 10.1038/292306a0

[msad167-B54] Janečka JE , Davis BW, Ghosh S, Paria N, Das PJ, Orlando L, Schubert M, Nielsen MK, Stout TAE, Brashear W, et al 2018. Horse Y chromosome assembly displays unique evolutionary features and putative stallion fertility genes. Nat Commun. 9:2945. 30054462 10.1038/s41467-018-05290-6 PMC6063916

[msad167-B55] Katoh K , Misawa K, Kuma K, Miyata T. 2002. MAFFT: a novel method for rapid multiple sequence alignment based on fast Fourier transform. Nucleic Acids Res. 30:3059–3066. 12136088 10.1093/nar/gkf436 PMC135756

[msad167-B56] Kaufmann P . 2022. The evolution of sexual dimorphism and its genetic underpinnings [doctoral thesis]. Uppsala: Acta Universitatis Upsaliensis Uppsala.

[msad167-B57] Kaufmann P , Howie JM, Immonen E. 2023. Sexually antagonistic selection maintains genetic variance when sexual dimorphism evolves. Proc R Soc. 290:20222484 10.1098/rspb.2022.2484 PMC10031426 36946115

[msad167-B58] Kaufmann P , Wolak ME, Husby A, Immonen E. 2021. Rapid evolution of sexual size dimorphism facilitated by Y-linked genetic variance. Nat Ecol Evol. 5:1394–1402. 34413504 10.1038/s41559-021-01530-z

[msad167-B59] Lampert KP , Schmidt C, Fischer P, Volff J-N, Hoffmann C, Muck J, Lohse MJ, Ryan MJ, Schartl M. 2010. Determination of onset of sexual maturation and mating behavior by melanocortin receptor 4 polymorphisms. Curr Biol. 20:1729–1734. 20869245 10.1016/j.cub.2010.08.029

[msad167-B60] Lande R . 1980. Sexual dimorphism, sexual selection, and adaptation in polygenic characters. Evolution 34:292–305. 28563426 10.1111/j.1558-5646.1980.tb04817.x

[msad167-B61] Larkin A , Marygold SJ, Antonazzo G, Attrill H, Dos Santos G, Garapati PV, Goodman JL, Gramates LS, Millburn G, Strelets VB, et al 2021. Flybase: updates to the *Drosophila melanogaster* knowledge base. Nucleic Acids Res. 49:D899–D907. 33219682 10.1093/nar/gkaa1026 PMC7779046

[msad167-B62] Lemos B , Araripe LO, Hartl DL. 2008. Polymorphic Y chromosomes harbor cryptic variation with manifold functional consequences. Science 319:91–93. 18174442 10.1126/science.1148861

[msad167-B63] Lenormand T , Fyon F, Sun E, Roze D. 2020. Sex chromosome degeneration by regulatory evolution. Curr Biol. 30:3001–3006.e5. 32559446 10.1016/j.cub.2020.05.052

[msad167-B64] Li H . 2018. Minimap2: pairwise alignment for nucleotide sequences. Bioinformatics 34:3094–3100. 29750242 10.1093/bioinformatics/bty191 PMC6137996

[msad167-B65] Liao Y , Smyth GK, Shi W. 2014. Featurecounts: an efficient general purpose program for assigning sequence reads to genomic features. Bioinformatics 30:923–930. 24227677 10.1093/bioinformatics/btt656

[msad167-B66] Lin MF. Rodeh O , Penn J, Bai X, Reid JG, Krasheninina O, Salerno WJ. 2018. GLnexus: joint variant calling for large cohort sequencing. bioRxiv 343970. 10.1101/343970.

[msad167-B67] Love MI , Huber W, Anders S. 2014. Moderated estimation of fold change and dispersion for RNA-Seq data with DESeq2. Genome Biol. 15:550. 10.1186/s13059-014-0550-8 PMC4302049 25516281

[msad167-B68] Mahajan S , Wei KH, Nalley MJ, Gibilisco L, Bachtrog D. 2018. De novo assembly of a young Drosophila Y chromosome using single-molecule sequencing and chromatin conformation capture. PLoS Biol. 2018;16(7):e2006348. 30059545 10.1371/journal.pbio.2006348 PMC6117089

[msad167-B69] Mank JE . 2012. Small but mighty: the evolutionary dynamics of W and y sex chromosomes. Chromosom Res. 20:21–33. 10.1007/s10577-011-9251-2 PMC3299550 22038285

[msad167-B70] Manni M , Berkeley MR, Seppey M, Simão FA, Zdobnov EM. 2021. BUSCO update: novel and streamlined workflows along with broader and deeper phylogenetic coverage for scoring of eukaryotic, prokaryotic, and viral genomes. Mol Biol Evol. 38:4647–4654. 34320186 10.1093/molbev/msab199 PMC8476166

[msad167-B71] McKenna DD , Shin S, Ahrens D, Balke M, Beza-Beza C, Clarke DJ, Donath A, Escalona HE, Friedrich F, Letsch H, et al 2019. The evolution and genomic basis of beetle diversity. Proc Natl Acad Sci U S A. 116:24729–24737. 31740605 10.1073/pnas.1909655116 PMC6900523

[msad167-B72] Murphy WJ , Wilkerson AJ, Raudsepp T, Agarwala R, Schäffer AA, Stanyon R, Chowdhary BP. 2006. Novel gene acquisition on carnivore Y chromosomes. PLoS Genet. 2:e43. 16596168 10.1371/journal.pgen.0020043 PMC1420679

[msad167-B73] Nielsen TM , Baldwin J, Fedorka KM. 2023. Gene-poor Y-chromosomes substantially impact male trait heritabilities and may help shape sexually dimorphic evolution. Heredity (Edinb). 130:236–241. 36759734 10.1038/s41437-023-00596-8 PMC10076275

[msad167-B74] Nursyifa C , Brüniche-Olsen A, Garcia-Erill G, Heller R, Albrechtsen A. 2022. Joint identification of sex and sex-linked scaffolds in non-model organisms using low depth sequencing data. Mol Ecol Resour. 22:458–467. 34431216 10.1111/1755-0998.13491

[msad167-B75] Paris JR , Whiting JR, Daniel MJ, Ferrer Obiol J, Parsons PJ, van der Zee MJ, Wheat CW, Hughes KA, Fraser BA. 2022. A large and diverse autosomal haplotype is associated with sex-linked colour polymorphism in the guppy. Nat Commun. 13:1233. 35264556 10.1038/s41467-022-28895-4 PMC8907176

[msad167-B76] Parker GA . 2006. Sexual conflict over mating and fertilization: an overview. Philos Trans R Soc B Biol Sci. 361:235–259. 10.1098/rstb.2005.1785 PMC1569603 16612884

[msad167-B77] Peichel CL , McCann SR, Ross JA, Naftaly AFS, Urton JR, Cech JN, Grimwood J, Schmutz J, Myers RM, Kingsley DM, et al 2020. Assembly of the threespine stickleback Y chromosome reveals convergent signatures of sex chromosome evolution. Genome Biol. 21:177. 32684159 10.1186/s13059-020-02097-x PMC7368989

[msad167-B78] Perry J , Kleckner N. 2003. The ATRs, ATMs, and TORs are giant HEAT repeat proteins. Cell 112:151–155. 12553904 10.1016/s0092-8674(03)00033-3

[msad167-B79] Poplin R , Chang P-C, Alexander D, Schwartz S, Colthurst T, Ku A, Newburger D, Dijamco J, Nguyen N, Afshar PT, et al 2018. A universal SNP and small-indel variant caller using deep neural networks. Nat Biotechnol. 36:983–987. 30247488 10.1038/nbt.4235

[msad167-B80] Prokop JW , Deschepper CF. 2015. Chromosome Y genetic variants: impact in animal models and on human disease. Physiol Genomics. 47:525–537. 26286457 10.1152/physiolgenomics.00074.2015 PMC4629007

[msad167-B81] Rhie A , Nurk S, Cechova M, Hoyt SJ, Taylor DJ, Altemose N, Hook PW, Koren S, Rautiainen M, Alexandrov IA, et al 2022. The complete sequence of a human Y chromosome. bioRxiv 518724. 10.1101/2022.12.01.518724.

[msad167-B82] Rice WR . 1984. Sex chromosomes and the evolution of sexual dimoprhism. Evolution 38:735–742. 28555827 10.1111/j.1558-5646.1984.tb00346.x

[msad167-B83] Rideout EJ , Narsaiya MS, Grewal SS. 2015. The sex determination gene transformer regulates male-female differences in Drosophila body size. PLoS Genet. 11:e1005683. 26710087 10.1371/journal.pgen.1005683 PMC4692505

[msad167-B84] Rogers TF , Palmer DH, Wright AE. 2021. Sex-specific selection drives the evolution of alternative splicing in birds. Mol Biol Evol. 38:519–530. 32977339 10.1093/molbev/msaa242 PMC7826194

[msad167-B85] Sandkam BA , Almeida P, Darolti I, Furman BLS, van der Bijl W, Morris J, Bourne GR, Breden F, Mank JE. 2021. Extreme Y chromosome polymorphism corresponds to five male reproductive morphs of a freshwater fish. Nat Ecol Evol. 5:939–948. 33958755 10.1038/s41559-021-01452-w

[msad167-B86] Sayadi A , Martinez Barrio A, Immonen E, Dainat J, Berger D, Tellgren-Roth C, Nystedt B, Arnqvist G. 2019. The genomic footprint of sexual conflict. Nat Ecol Evol. 3:1725–1730. 31740847 10.1038/s41559-019-1041-9

[msad167-B87] Schärer L , Rowe L, Arnqvist G. 2012. Anisogamy, chance and the evolution of sex roles. Trends Ecol Evol. 27:260–264. 22277154 10.1016/j.tree.2011.12.006

[msad167-B88] Shao X , Lv N, Liao J, Long J, Xue R, Ai N, Xu D, Fan X. 2019. Copy number variation is highly correlated with differential gene expression: a pan-cancer study. BMC Med Genet. 20:175. 31706287 10.1186/s12881-019-0909-5 PMC6842483

[msad167-B89] Singh P , Taborsky M, Peichel CL, Sturmbauer C. 2023. Genomic basis of Y-linked dwarfism in cichlids pursuing alternative reproductive tactics. Mol Ecol. 32:1592–1607 36588349 10.1111/mec.16839

[msad167-B90] Skaletsky H , Kuroda-Kawaguchi T, Minx PJ, Cordum HS, Hillier L, Brown LG, Repping S, Pyntikova T, Ali J, Bieri T, et al 2003. The male-specific region of the human Y chromosome is a mosaic of discrete sequence classes. Nature 423:825–837. 12815422 10.1038/nature01722

[msad167-B91] Slater GSC , Birney E. 2005. Automated generation of heuristics for biological sequence comparison. BMC Bioinformatics. 6:31. 15713233 10.1186/1471-2105-6-31 PMC553969

[msad167-B92] Smeds L , Warmuth V, Bolivar P, Uebbing S, Burri R, Suh A, Nater A, Bureš S, Garamszegi LZ, Hogner S, et al 2015. Evolutionary analysis of the female-specific avian W chromosome. Nat Commun. 6:7330. 26040272 10.1038/ncomms8330 PMC4468903

[msad167-B93] Smit AFA , Hubley R, Green P. 2015. RepeatMasker Open 4.0.

[msad167-B94] Soh YQS , Alföldi J, Pyntikova T, Brown LG, Graves T, Minx PJ, Fulton RS, Kremitzki C, Koutseva N, Mueller JL, et al 2014. Sequencing the mouse Y chromosome reveals convergent gene acquisition and amplification on both sex chromosomes. Cell 159:800–813. 25417157 10.1016/j.cell.2014.09.052 PMC4260969

[msad167-B95] Swer PB , Mishra H, Lohia R, Saran S. 2016. Overexpression of TOR (target of rapamycin) inhibits cell proliferation in *Dictyostelium discoideum*. J Basic Microbiol. 56:510–519. 26460541 10.1002/jobm.201500313

[msad167-B96] Vilella-Bach M , Nuzzi P, Fang Y, Chen J. 1999. The FKBP12-rapamycin-binding domain is required for FKBP12-rapamycin- associated protein kinase activity and G1 progression. J Biol Chem. 274:4266–4272. 9933627 10.1074/jbc.274.7.4266

[msad167-B97] Wu TD , Watanabe CK. 2005. GMAP: a genomic mapping and alignment program for mRNA and EST sequences. Bioinformatics 21:1859–1875. 15728110 10.1093/bioinformatics/bti310

[msad167-B98] Wullschleger S , Loewith R, Hall MN. 2006. TOR Signaling in growth and metabolism. Cell 124:471–484. 16469695 10.1016/j.cell.2006.01.016

[msad167-B99] Zhang J , Luo J, Chen J, Dai J, Montell C. 2020. The role of Y chromosome genes in male fertility in *Drosophila melanogaster*. Genetics 215:623–633. 32404399 10.1534/genetics.120.303324 PMC7337068

[msad167-B100] Zhou S , Yang Y, Scott MJ, Pannuti A, Fehr KC, Eisen A, Koonin EV, Fouts DL, Wrightsman R, Manning JE. 1995. Male-specific lethal 2, a dosage compensation gene of Drosophila, undergoes sex-specific regulation and encodes a protein with a RING finger and a metallothionein-like cysteine cluster. EMBO J. 14:2884–2895. 7796814 10.1002/j.1460-2075.1995.tb07288.x PMC398407

